# Associations Between Vertebral Marrow Proton Density Fat Fraction and Risk of Prostate Cancer

**DOI:** 10.3389/fendo.2022.874904

**Published:** 2022-04-14

**Authors:** Shaojun Li, Bo Wang, Wenwen Liang, Qi Chen, Wei Wang, Jiangjun Mei, He Zhang, Qianqian Liu, Mingyuan Yuan

**Affiliations:** ^1^ Department of Radiology, Shanghai University of Medicine & Health Sciences Affiliated Zhoupu Hospital, Shanghai, China; ^2^ Department of Radiology, Gongli Hospital of Shanghai Pudong New Area, Shanghai, China; ^3^ Department of Ultrasound Medicine, Shanghai University of Medicine & Health Sciences Affiliated Zhoupu Hospital, Shanghai, China; ^4^ Department of Urology, Gongli Hospital of Shanghai Pudong New Area, Shanghai, China; ^5^ Department of Laboratory Medicine, Gongli Hospital of Shanghai Pudong New Area, Shanghai, China

**Keywords:** prostate cancer risk, bone marrow, adipocyte, proton density fat fraction, MR spectroscopy

## Abstract

Bone marrow adipocytes may be responsible for cancer progression. Although marrow adipogenesis is suspected to be involved in prostate carcinogenesis, an association between marrow adiposity and prostate cancer risk has not been clearly established *in vivo*. This work included 115 newly diagnosed cases of histologically confirmed prostate cancer (range, 48–79 years) and 87 age-matched healthy controls. Marrow proton density fat fraction (PDFF) was measured by 3.0-T MR spectroscopy at the spine lumbar. Associations between marrow PDFF and risk of prostate cancer by stage of disease and grade sub-types were performed using multivariable polytomous logistic regression. There were no significant group differences in the vertebral marrow PDFF, despite prostate cancer patients having 6.6% higher marrow PDFF compared to the healthy controls (61.7 ± 9.8% vs. 57.9 ± 6.5%; *t* = 1.429, *p* = 0.161). After adjusting for various clinical and demographic characteristics, we found that elevated marrow PDFF was related to an increased risk of high-grade prostate cancer [odds ratios (OR) = 1.31; 95% confidence interval (CI), 1.08–1.57; *p* = 0.003]. Likewise, increased marrow PDFF had a significantly positive correlation with aggressive prostate cancer risk (OR = 1.54; 95% CI, 1.13–1.92; *p <*0.001). There were no associations between marrow PDFF and low-grade (*p* = 0.314) or non-aggressive (*p* = 0.435) prostate cancer risk. The data support the hypothesis that marrow adiposity was correlated with increased risk of aggressive prostate cancer, supporting a link between adipogenesis and prostate cancer risk.

## Introduction

Marrow adipocytes are derived from multipotent bone marrow mesenchymal stem cells that can differentiate into various cell types, including myocytes, osteoblasts, chondrocytes, and adipocytes (1). Adipocytes within the bone marrow niche carry out diverse functions that are capable of secreting inflammatory mediators, growth factors, and adipokines, which play an active role in regulating the function and behavior of neighboring cells, and have potentials to dysregulate normal bone homeostasis (2–4).

The unique features of the bone marrow niche make it a favorable metastatic site of cancers, including multiple myeloma, prostate, and breast cancer bone metastasis (4–6). The positive feedback loops initiated by tumor cells within the bone marrow niche also induce these cancer cells to metastasize and to grow within the bone marrow but not in other anatomical sites (7). Mechanisms resulting in the homing of prostate cancer cells to bone marrow remain poorly understood. Several studies have demonstrated that marrow fat tissue fuels and supports the growth of solid tumor metastasis, such as breast and prostate cancer bone metastasis, by serving as plenty of source of lipids and signaling molecules (4, 8). As a powerful tool to reveal novel mechanistic targets of cancer-related bone-metastatic diseases treatments, understanding the prominent role of marrow adiposity in adaptation and survival of tumor cells in the bone is of great clinical implication. Skeletal metastases are a major cause of morbidity and mortality in prostate cancer.

Skeletal metastasis remains the most common and the deadliest complication of advanced prostate cancer. The role of marrow fat cells in the bone metastatic process of prostate cancer cells is well established (9, 10). *Ex vivo* human primary marrow adipocyte secretions may support prostate-cancer-directed migration in a chemokine receptor CCR3-dependent manner (5), whereas the number and depth of *in vivo* analyses in patients are limited. We hypothesized that an increase in marrow proton density fat fraction measured by magnetic resonance spectroscopy (MRS) in spinal vertebral bodies may be correlated with increased prostate cancer risk, both overall and subgroup according to stage and grade. If this is the case, marrow adiposity may present a promising therapeutic option for aggressive prostate cancer.

## Materials and Methods

### Study Population

This study includes 115 newly diagnosed, histologically confirmed, primary prostate cancer (range, 48–79 years) identified by the Pathology Department between December 2017 and November 2021. We also recruited 87 age-matched healthy controls (range, 49–78 years) from the community on the basis of the participant’s age at the date of the diagnosis. Prostate cancer patients were classified into localized (stage T1 or T2; NXM0) or advanced stage (stage T3 or T4) according to the tumor–node–metastasis (TNM) clinical staging system and into low grade (Gleason score <7) and high grade (Gleason sum ≥7) according to Gleason scores following review of histological material, as previously described (11). Aggressive disease was classified by TNM stage III–IV and/or Gleason sum 7 or higher. Non-aggressive disease was defined as Gleason score <7 and stage I/II at diagnosis (12).

At enrollment, all patients completed self-administered questionnaires about demographics, general risk factors, smoking status, and medical history, family history of prostate cancer, and lifestyle factors (e.g., physical activity, history of smoking, and alcohol consumption). Cigarette smoking at baseline was classified as current, former, or never. Physical activity was determined by using the International Physical Activity Questionnaire short form, with data reported as Metabolic Equivalent of Task hours per week (6). Height was measured to the nearest 0.5 cm with a metric rule attached to the wall and a right angled wood block. Body weight was measured to the nearest 0.1 kg with a balance beam scale after participants removed footwear and excess clothing. Body mass index was calculated as the weight (in kilograms) divided by the square of the height (in meters). Participants also had an overnight fasting blood sample collected. Participants were excluded in the following cases: (1) previous cancer or another history of chronic illness such as diabetes mellitus, hypothalamic or pituitary disorders, or impaired renal function; (2) use of medications known to influence bone metabolism such as bisphosphonates, steroids, hormone replacement therapy, or any medications that affect bone metabolism; (3) previous history of lumbar spinal surgery or irradiation; and (4) any contraindication to MR examinations. The study protocols conformed to the Declaration of Helsinki. The study was approved by the Institutional Review Board of Shanghai University of Medicine & Health Sciences Affiliated Zhoupu Hospital, and all participants signed a letter of informed consent for the scientific evaluation of their data.

### Biochemical Analyses

All blood samples were obtained before 8 a.m. after an 8-h overnight fast and were analyzed on the same day as collection. The plasma concentration of glucose was determined using an automatic biochemical analyzer (ADVIA Chemistry XPT System, Siemens Healthcare, Erlangen, Germany). Prostate-specific antigen, total cholesterol, triglycerides, high- and low-density lipoprotein cholesterol, and total cholesterol were measured with a Cobas 8000 (Roche Diagnostics, Basel, Switzerland) using chemiluminescence immunoassay.

### MRI Examinations

For marrow proton density fat fraction (PDFF) measurements of the L3 vertebra, the participants underwent spine ^1^H-magnetic resonance spectroscopy (^1^H-MRS) in a 3-T MRI instrument (Siemens Skyra, Siemens Medical Systems, Erlangen, Germany), as previously described (13, 14). Briefly, the participants were positioned head first in the magnet bore in the prone position. A phased-array coil was positioned over the lumbar region. To exclude any confounders such as silent compression fractures, vertebral hemangiomas, and wedging of vertebrae, the standard protocols including sagittal T1- and T2-weighted images were used for anatomical and morphological evaluations of the lumbar spine. Before the MR spectroscopy acquisition, coronal, sagittal, and axial scout T2-weighted fast spin echo sequence were used as a reference for the spectral acquisition box.

**Figure 1:**
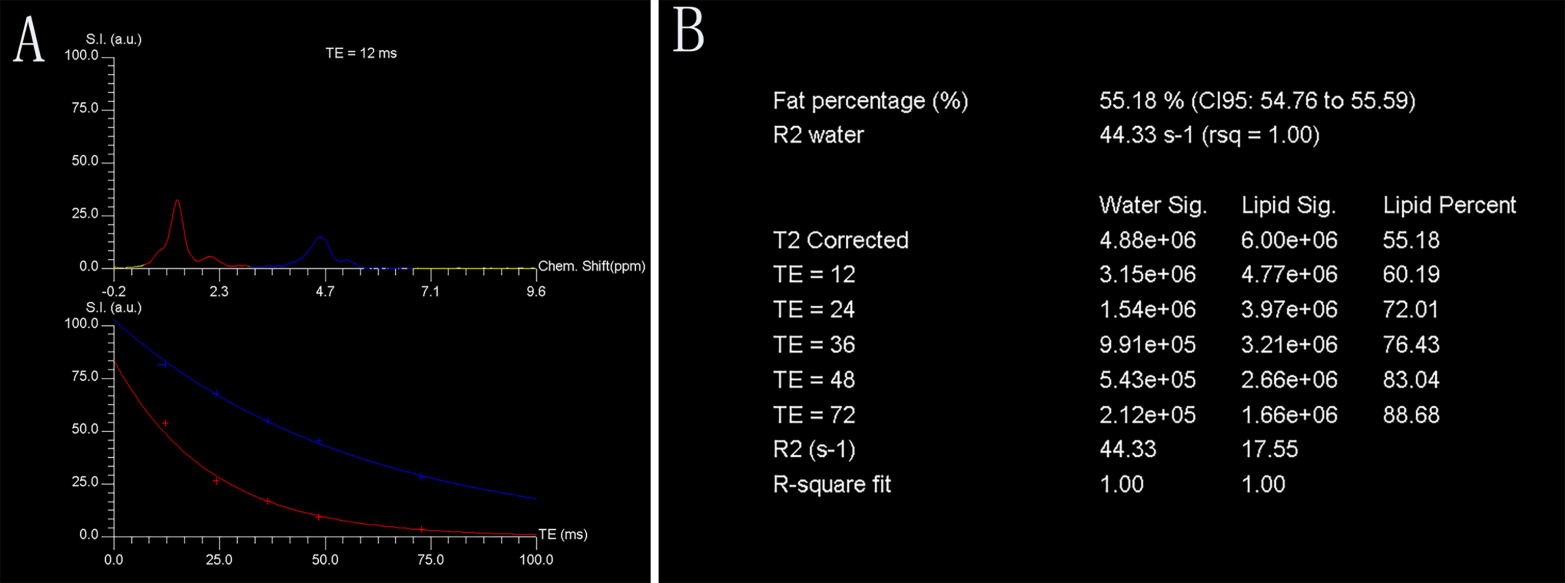
Representative single-voxel T2-corrected multi-echo magnetic resonance spectroscopy. Fat and water spectral peaks at echo time of 12 ms are presented. Red curve denotes fat spectrum (bulk methylene protons at 1.30 ppm), and blue curve denotes water spectrum (H_2_O at 4.7 ppm) **(A)**. Each fat and water integral at five echoes (echo time = 12, 24, 36, 48, and 72 ms, respectively) with estimated marrow PDFF of 55.18% **(B)**. Chem., chemical; CI, confidence interval; FF, fat fraction; rsq, r-squared; S.I. (a.u.), signal intensity (arbitrary units); TE, echo time.

A single voxel T2-corrected multi-echo MR spectroscopy sequence was performed by placing a voxel measuring 1.5 mm × 1.5 mm × 1.5 mm (3.4 ml) within the L3 vertebral body using a stimulated echo acquisition mode with five echo times (12, 24, 36, 48, and 72 ms) and 3,000 ms repetition time. Spectral data were acquired in 1,024 data points with a 2,000 Hz receiver bandwidth, 90° flip angle without water suppression, and a total acquisition time of 17 s. For each voxel placement, automated procedures were performed to optimize the gradient shimming and transmit and receive gain. An example of the fat spectral and water peaks at a specific echo time and the automatically calculated fat fraction from the high-speed T2-corrected multi-echo scan are displayed in **Figure 1**.

### Statistical Analysis

Data are expressed as the mean ± standard deviation, median (interquartile range), or frequencies and percentages where appropriate. Differences in baseline demographic and clinical characteristics between cases and healthy controls were evaluated using *t*-test or Wilcoxon rank sum test (for continuous variables) and chi-squared tests (for categorical variables). Differences in the PDFF values among the controls and low- and high-grade prostate cancer, and among the controls and aggressive and non-aggressive prostate cancer were assessed using analysis of covariance (ANCOVA) followed by Bonferroni *post-hoc* multiple comparisons when controlling for covariates. Multivariable polytomous logistic regression was used to estimate odds ratios (ORs) and 95% confidence intervals (CIs) for the associations of marrow PDFF with prostate cancer risk by stage and grade sub-types, in which two separate sets of analyses were ran. The potential confounding factors that are hypothesized or known to be associated with prostate cancer were included in the multivariable models including age, body mass index (BMI), physical activity, alcohol consumption, smoking status, levels of prostate specific antigen and blood lipids, and family history of prostate cancer (15, 16). These statistical analyses were performed using IBM SPSS statistical software version 20 (SPSS Inc., Chicago, IL, USA). All statistical tests were two sided, and *p*-values were considered to be significant if <0.05.

## Results

### General Outcomes

The demographic and clinical characteristics of the prostate cancer patients and healthy controls are presented in **Table 1**. Among the total 115 prostate cancer, 60 (52.2%) were classified as aggressive prostate cancer, and 52 (45.2%) had a Gleason score ≥7. Cases and healthy controls did not differ markedly in demographic characteristics. Prostate cancer patients had higher levels of prostate-specific antigen than healthy controls (median, 10.2 vs. 2.1 ng/ml; *p* < 0.001) and were more likely to have hypercholesterolemia. There were no other differences between groups in family history of prostate cancer, smoking status, alcohol intake, and levels of physical activity. There were no significant group differences in the marrow PDFF at the spine lumbar, despite prostate cancer patients having 6.6% higher marrow PDFF compared to the healthy controls (61.7 ± 9.8% vs. 57.9 ± 6.5%; *t* = 1.429, *p* = 0.161) (**Table 1**).

**Table 1:** Baseline characteristics of the study population.

	Prostate cancer (n = 115)	Healthy controls (n = 87)
Age, years	61.6 ± 8.8	62.5 ± 8.6
Height, cm	170.5 ± 7.1	171.0 ± 8.5
Weight, kg	62.9 ± 8.9	63.1 ± 9.0
BMI, kg/m^2^	23.9 ± 4.2	23.5 ± 3.9
Family history of prostate cancer, n (%)	6 (5.2)	2 (2.3)
Smoking status, n (%)		
Never	75 (65.2)	60 (69.0)
Former smoker	25 (21.7)	17 (19.5)
Current smoker	15 (13.0)	10 (11.5)
Alcohol intake, n (%)		
None	87 (75.7)	68 (78.2)
≤7 units per week	19 (16.5)	13 (14.9)
>7 units per week	9 (7.8)	6 (6.9)
Prostate-specific antigen, ng/ml	10.2 (6.1, 28.5)	2.1 (1.0, 7.6) ^a^
Histologic grade, n (%)		
Low (Gleason sum: 2–6)	63 (54.8)	NA
High (Gleason sum: ≥7)	52 (45.2)	NA
Stage of disease, n (%)		
I/II	79 (68.7)	NA
III/IV	36 (31.3)	NA
Physical activity, METs/week	9 (4, 16)	10 (4, 18)
Total cholesterol, mmol/L	4.62 (4.28, 4.96)	4.11 (3.98, 4.33) ^a^
Triglyceride, mmol/L	1.29 (1.24, 1.34)	1.26 (1.19, 1.30)
HDL cholesterol, mmol/L	1.27 (1.23, 1.31)	1.28 (1.25, 1.34)
LDL cholesterol, mmol/L	2.97 (2.83, 3.12)	2.82 (2.68, 3.23)
PDFF, %	61.7 ± 9.8	57.9 ± 6.5 ^b^

Data are expressed as mean ± SD, median (IQR), or n (%) as appropriate.

BMI, body mass index; HDL, high-density lipoprotein; IQR, interquartile range Q1-Q3; LDL, low-density lipoprotein; METs, metabolic equivalent of tasks; NA, not applicable; SD, standard deviation; PDFF, proton density fat fraction.

To detect difference between the two groups.

a p value by Wilcoxon rank sum test.

b p value by independent-sample t-test (all p<0.05).

### Differences in PDFF Among the Controls and Low- and High-Grade Prostate Cancer, and Among the Controls and Aggressive and Non-aggressive Prostate Cancer

**Figure 2:**
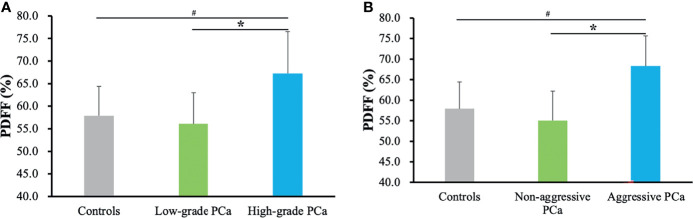
Marrow PDFF results from the healthy controls, low- and high-grade prostate cancer **(A)**, and aggressive and non-aggressive prostate cancer **(B)**. Data are expressed as mean ± SD. ^#^ *p* < 0.001 and *p < 0.001 were calculated by ANCOVA followed by Bonferroni *post-hoc* multiple comparisons after adjusting for age, body mass index, alcohol intake, smoking status, physical activity, prostate specific antigen levels, levels of blood lipids, and family history of prostate cancer. PCa, prostate cancer; PDFF, proton density fat fraction.

**Figure 2** summarizes the marrow PDFF results from the healthy controls, low- and high-grade prostate cancer, and aggressive and non-aggressive prostate cancer. In the unadjusted models, lumbar spine PDFF was higher in the high-grade prostate cancer patients (67.3 ± 9.2%) than those in health controls (57.9 ± 6.5%, *p* < 0.001) or low-grade prostate cancer (56.1 ± 6.9%, *p* < 0.001), and these differences remained statistically significant even after controlling for various clinical and demographic characteristics including age, body mass index, smoking status, alcohol intake, physical activity, prostate specific antigen levels, levels of blood lipids, and family history of prostate cancer. Similar results were observed between the healthy controls (57.9 ± 6.5%) and aggressive prostate cancer patients (68.3 ± 7.4%, *p* < 0.001), and between the non-aggressive (55.0 ± 7.2%, *p* < 0.001) and aggressive prostate cancer patients (68.3 ± 7.4%, *p* < 0.001) (**Figure 2**).

### Relationships Between Proton Density Fat Fraction and Prostate Cancer Risk

**Table 2** gives multivariable-adjusted associations of marrow PDFF with risk of low- and high-grade prostate cancer and non-aggressive and aggressive prostate cancer, respectively. When stratified by Gleason scores, there were significant associations between elevated marrow PDFF and risk of low- or high-grade prostate cancer in the univariable analysis (all *p* < 0.05). Similar findings were observed in non-aggressive and aggressive disease.

**Table 2:** Association between marrow proton density fat fraction and prostate cancer risk.

	Unadjusted		Multivariable adjusted*	
	OR (95% CI)	*p*-value	OR (95% CI)	*p*-value
Low-grade prostate cancer	1.35 (1.09–1.55)	0.017	1.19 (0.88–1.49)	0.314
High-grade prostate cancer	1.47 (1.16–1.80)	<0.001	1.31 (1.08–1.57)	0.003
Non-aggressive prostate cancer	1.40 (1.08–1.69)	0.021	1.29 (0.90–1.77)	0.435
Aggressive prostate cancer	1.61 (1.20–2.04)	<0.001	1.54 (1.13−1.92)	<0.001

Low-grade was classified by Gleason score <7, and high-grade was classified by Gleason sum ≥7 according to Gleason scores. Aggressive disease was classified by Gleason grade ≥ 7 and/or stage III–IV, and non-aggressive disease was defined as Gleason score <7 and stage I/II at diagnosis.

^*^Multivariable adjusted for age, body mass index, smoking status, alcohol use, physical activity, prostate specific antigen, levels of blood lipids, and family history of prostate cancer.

OR, odds ratio; CI, confidence interval.

In multivariable analysis adjusted for aforementioned covariates, increased marrow PDFF was correlated with an increased risk of high-grade prostate cancer (OR = 1.31; 95% CI, 1.08–1.57; *p* = 0.003), but this relationship did not reach significance in risk of low-grade prostate cancer (OR = 1.19; 95% CI, 0.88–1.49; *p* = 0.314). Similar results were found in aggressive prostate cancer risk (OR = 1.54; 95% CI, 1.13–1.92; *p <*0.001) but not in non-aggressive prostate cancer risk (**Table 2**).

## Discussion

Bone marrow niche is dynamic and complex. The functional role of bone marrow fat cells in the development and progression, phenotype, and survival of skeletal tumor cells remains elusive. Adipocyte-enriched microenvironments is a preferred site for prostate cancer bone metastasis, and several studies have focused on the cellular and molecular mechanisms of this tropism, indicating that a complex interaction occurs between neoplastic cells and the cells directly responsible for skeletal remodeling, namely, osteoclasts and osteoblasts in modulating the microenvironment of bone marrow (17). Expansion of marrow adiposity has shown to be related with both osteoblastic and osteolytic disease (9, 18). We found that prostate cancer patients were more likely to have a higher marrow PDFF. Our data in the present study may provide *in vivo* imaging evidence supporting previous *in vitro* studies, indicating that in multiple myeloma, breast, and prostate cancer, crosstalk of adipocytes–tumor cells is one of the important mediators for implantation and propagation of the migrating metastatic cells and may serve as an emerging facilitator of therapy evasion (19, 20). Therapeutic approaches that modulate the balance between osteogenesis and adipogenesis may be one of the most effective ways to maintain healthy skeletal integrity, thus preventing and treating cancer infiltration.

Overall, in this large retrospective study, we reported an odds ratio of 1.31−1.54 for marrow fat fraction to predict high-grade or aggressive prostate cancer on multivariable analysis, suggesting a possible link between expansion of marrow fat and greater risk of high-grade disease. Several mechanisms could explain the associations of marrow adiposity with prostate cancer risk. The representative mechanisms include the secretion of adipocytokines and lipid transfer (21). Previous studies have shown that prostate cancers cells are attracted to a rich source of lipids stored within adipocytes in the bone marrow (22). In the bone marrow, fat cells are indeed active cells because these cells actively not only store free fatty acids but also secrete chemokines, adipokines, growth factors, and inflammatory mediators such as interleukin 1 beta, interleukin 6, and tumor necrosis factor alpha, which affect bone remodeling, insulin metabolism, and energy regulation (23, 24). Increased marrow adiposity is tightly linked to marrow inflammation. In various physiological conditions, inflammatory pathways may maintain normal bone metabolism but are significantly altered in condition of marrow fat expansion (10). For example, bone marrow fat cells secrete significant and regulated levels of IL-6, which promotes metastasis of tumor cells through the JAK2/STAT3 signaling pathway (24, 25). Upregulated expression of interleukin 6 in malignant cells was found to promote osteoclastogenesis. Similarly, several studies have linked marrow fat cells-derived CXCL1 and CXCL2 chemokines with prostate tumor progression and the effects of osteolysis in metastatic prostate cancer (9, 10, 26).

Furthermore, adipocytes’ impact on the tumor cell behavior may attribute to the regulation of cancer cell metabolism *via* lipid transfer and lipolysis (4). The number of marrow adipocytes was increased in a metastatic bone marrow niche at the early phase; interestingly, during tumor progression, the number of fat cells with abundant lipid droplets was decreased (21, 27). These data provide *in vivo* evidence supporting the essential role of adipocytes in the lipid transfer from marrow fat cells to tumor cells. Previous *ex vivo* and *in vivo* studies also have shown that lipid chaperone fatty acid-binding protein 4 and interleukin 1 beta expression in metastatic prostate carcinoma cells are markedly induced by the exposure to bone marrow adipocytes in a functional crosstalk (8, 10). Diedrich et al. provided evidence that marrow adipocytes are able to induce the glycolytic phenotypes in prostate cancer cells by paracrine regulation of glycolytic enzymes, increasing lactate production and decreasing mitochondrial oxidative phosphorylation (28). They also found that fat cells are capable of driving metabolic reprogramming of metastatic prostate tumors through oxygen-independent mechanism of activating hypoxia-inducible factor 1 alpha signaling that can be reversed by hypoxia-inducible factor 1 alpha downregulation. Based on these data, targeting lipid metabolism of marrow fat cells combined with standard therapeutic agents may present unique therapeutic protocols for some cancers that thrive in adipocyte-rich metabolically active red bone marrow.

There are several strengths in our study, including its primary strength, its relatively large sample size, which allowed us to determine associations with low- and high-grade prostate cancer risk. Second, we uniformly used the Gleason scoring system to assess all tumors, which minimizes the large intra-rater variability in assigning clinical grade. Third, the precise marrow fat content at spinal vertebral bodies was obtained by using MR spectroscopy *in vivo*. The limitations of our study also must be acknowledged. First, the cross-sectional design of the study indicates that causality cannot be established for the outcomes assessed. Second, our study consisted of only Chinese men, which may limit the generalizability of our findings to men in other racial/ethnic groups. Future studies should focus on the relationship in other populations. Furthermore, although detailed evaluations of several lifestyle factors enabled controlling for confounding factors, residual or unmeasured confounding factors such as individual bone mineral density, caffeine or tea intake, and calcium and vitamin D supplementation cannot be ruled out.

In conclusion, our findings showed a close link between marrow adiposity and an increased risk of high-grade or aggressive prostate cancer but did not support an association between marrow adiposity and risk of low-grade or non-aggressive prostate cancer. MRI plays a critical role in the non-invasive assessment of bone marrow composition. Measures of marrow fat content may be predictive of prostate cancer risk. Future studies should specifically assess the associations of the diversity and complexity of bone marrow adipocytes with risk of prostate cancer, peculiarly prostate cancer bone metastasis.

## Data Availability Statement

The original contributions presented in the study are included in the article/supplementary material, further inquiries can be directed to the corresponding author.

## Ethics Statement

The studies involving human participants were reviewed and approved by the Institutional Review Board of Shanghai University of Medicine & Health Sciences Affiliated Zhoupu Hospital. The patients/participants provided their written informed consent to participate in this study.

## Author Contributions

Study design: SL, BW, and MY. Study conduct: JM, HZ, and QL. Data collection: WL, QC, and WW. Data analysis: SL, QC, and JM. Data interpretation: SL, BW, WL, QL, and MY. Drafting manuscript: SL, BW, and WL. All authors contributed to the article and approved the submitted version.

## Funding

This work was supported by the funds for Training Project of Academic Leaders of Health System in Pudong New Area of Shanghai (No. PWRd2021-07) and 2020 New Interdisciplinary Construction Project of Shanghai Pudong New Area Health (No. PWXx2020-06).

## Conflict of Interest

The authors declare that the research was conducted in the absence of any commercial or financial relationships that could be construed as a potential conflict of interest.

## Publisher’s Note

All claims expressed in this article are solely those of the authors and do not necessarily represent those of their affiliated organizations, or those of the publisher, the editors and the reviewers. Any product that may be evaluated in this article, or claim that may be made by its manufacturer, is not guaranteed or endorsed by the publisher.

## References

[1] Zayzafoon M Gathings WE McDonald JM. Modeled Microgravity Inhibits Osteogenic Differentiation of Human Mesenchymal Stem Cells and Increases Adipogenesis. Endocrinology (2004) 145:2421–32. doi: 10.1210/en.2003-1156 14749352

[2] Pierce JL Begun DL Westendorf JJ McGee-Lawrence ME. Defining Osteoblast and Adipocyte Lineages in the Bone Marrow. Bone (2018) 118:2–7. doi: 10.1016/j.bone.2018.05.019 29782940 PMC6240509

[3] Wang H Leng Y Gong Y. Bone Marrow Fat and Hematopoiesis. Front Endocrinol (Lausanne) (2018) 9:694. doi: 10.3389/fendo.2018.00694 30546345 PMC6280186

[4] Herroon MK Diedrich JD Podgorski I. New 3D-Culture Approaches to Study Interactions of Bone Marrow Adipocytes With Metastatic Prostate Cancer Cells. Front Endocrinol (Lausanne) (2016) 7:84. doi: 10.3389/fendo.2016.00084 27458427 PMC4933721

[5] Guerard A Laurent V Fromont G Esteve D Gilhodes J Bonnelye E. The Chemokine Receptor CCR3 Is Potentially Involved in the Homing of Prostate Cancer Cells to Bone: Implication of Bone-Marrow Adipocytes. Int J Mol Sci (2021) 22(4):1994 doi: 10.3390/ijms22041994 33671469 PMC7922974

[6] Li G Xu Z Zhuang A Chang S Hou L Chen Y. Magnetic Resonance Spectroscopy-Detected Change in Marrow Adiposity Is Strongly Correlated to Postmenopausal Breast Cancer Risk. Clin Breast Cancer (2017) 17:239–44. doi: 10.1016/j.clbc.2017.01.004 28188108

[7] Reagan MR Rosen CJ. Navigating the Bone Marrow Niche: Translational Insights and Cancer-Driven Dysfunction. Nat Rev Rheumatol (2016) 12:154–68. doi: 10.1038/nrrheum.2015.160 PMC4947935 26607387

[8] Herroon MK Diedrich JD Rajagurubandara E Martin C Maddipati KR Kim S. Prostate Tumor Cell-Derived IL-1β Induces an Inflammatory Phenotype in Bone Marrow Adipocytes and Reduces Sensitivity to Docetaxel *via* Lipolysis-Dependent Mechanisms. Mol Cancer Res (2019) 17:2508–21. doi: 10.1158/1541-7786.MCR-19-0540 PMC6891205 31562254

[9] Hardaway AL Herroon MK Rajagurubandara E Podgorski I. Marrow Adipocyte-Derived CXCL1 and CXCL2 Contribute to Osteolysis in Metastatic Prostate Cancer. Clin Exp Metastasis (2015) 32:353–68. doi: 10.1007/s10585-015-9714-5 PMC4393805 25802102

[10] Hardaway AL Herroon MK Rajagurubandara E Podgorski I. Bone Marrow Fat: Linking Adipocyte-Induced Inflammation With Skeletal Metastases. Cancer Metastasis Rev (2014) 33:527–43. doi: 10.1007/s10555-013-9484-y PMC4154371 24398857

[11] Zuccolo L Lewis SJ Donovan JL Hamdy FC Neal DE Smith GD. Alcohol Consumption and PSA-Detected Prostate Cancer Risk–A Case-Control Nested in the ProtecT Study. Int J Cancer (2013) 132:2176–85. doi: 10.1002/ijc.27877 PMC3786564 23024014

[12] Bonn SE Barnett MJ Thornquist M Goodman G Neuhouser ML. Body Mass Index and Prostate Cancer Risk in the Carotene and Retinol Efficacy Trial. Eur J Cancer Prev (2019) 28:212–9. doi: 10.1097/cej.0000000000000438 PMC6128789 29521683

[13] Li G Xu Z Lin H Chen Y Li X Chang S. Association Between Insulin Resistance and the Magnetic Resonance Spectroscopy-Determined Marrow Fat Fraction in Nondiabetic Postmenopausal Women. Menopause (2018) 25:676–82. doi: 10.1097/GME.0000000000001063 29360704

[14] Moorthi RN Fadel W Eckert GJ Ponsler-Sipes K Moe SM Lin C. Bone Marrow Fat is Increased in Chronic Kidney Disease by Magnetic Resonance Spectroscopy. Osteoporos Int (2015) 26:1801–7. doi: 10.1007/s00198-015-3064-7 PMC4582653 25701052

[15] Mondul AM Weinstein SJ Bosworth T Remaley AT Virtamo J Albanes D. Circulating Thyroxine, Thyroid-Stimulating Hormone, and Hypothyroid Status and the Risk of Prostate Cancer. PloS One (2012) 7:e47730. doi: 10.1371/journal.pone.0047730 23118893 PMC3484141

[16] Jamnagerwalla J Howard LE Allott EH Vidal AC Moreira DM Castro-Santamaria R. Serum Cholesterol and Risk of High-Grade Prostate Cancer: Results From the REDUCE Study. Prostate Cancer Prostatic Dis (2018) 21:252–9. doi: 10.1038/s41391-017-0030-9 PMC6021229 29282360

[17] Vičić I Belev B. The Pathogenesis of Bone Metastasis in Solid Tumors: A Review. Croatian Med J (2021) 62:270–82. doi: 10.3325/cmj.2021.62.270 PMC8275949 34212564

[18] Singh L Brennan TA Russell E Kim JH Chen Q Brad Johnson F. Aging Alters Bone-Fat Reciprocity by Shifting *In Vivo* Mesenchymal Precursor Cell Fate Towards an Adipogenic Lineage. Bone (2016) 85:29–36. doi: 10.1016/j.bone.2016.01.014 26805026 PMC4792752

[19] Fraczak E Olbromski M Piotrowska A Glatzel-Plucinska N Dziegiel P Dybko J. Bone Marrow Adipocytes in Haematological Malignancies. Acta Histochem (2018) 120:22–7. doi: 10.1016/j.acthis.2017.10.010 29146005

[20] Nieman KM Kenny HA Penicka CV Ladanyi A Buell-Gutbrod R Zillhardt MR. Adipocytes Promote Ovarian Cancer Metastasis and Provide Energy for Rapid Tumor Growth. Nat Med (2011) 17:1498–503. doi: 10.1038/nm.2492 PMC4157349 22037646

[21] Cha YJ Koo JS. Roles of Omental and Bone Marrow Adipocytes in Tumor Biology. Adipocyte (2019) 8:304–17. doi: 10.1080/21623945.2019.1643189 PMC6768257 31334678

[22] Gazi E Gardner P Lockyer NP Hart CA Brown MD Clarke NW. Direct Evidence of Lipid Translocation Between Adipocytes and Prostate Cancer Cells With Imaging FTIR Microspectroscopy. J Lipid Res (2007) 48:1846–56. doi: 10.1194/jlr.M700131-JLR200 17496269

[23] Wang D Haile A Jones LC. Dexamethasone-Induced Lipolysis Increases the Adverse Effect of Adipocytes on Osteoblasts Using Cells Derived From Human Mesenchymal Stem Cells. Bone (2013) 53:520–30. doi: 10.1016/j.bone.2013.01.009 23328495

[24] Laharrague P Fontanilles AM Tkaczuk J Corberand JX Pénicaud L Casteilla L. Inflammatory/Haematopoietic Cytokine Production by Human Bone Marrow Adipocytes. Eur Cytokine Netw (2000) 11:634–9. 11125307

[25] Wang L Cao L Wang H Liu B Zhang Q Meng Z. Cancer-Associated Fibroblasts Enhance Metastatic Potential of Lung Cancer Cells Through IL-6/STAT3 Signaling Pathway. Oncotarget (2017) 8:76116–28. doi: 10.18632/oncotarget.18814 PMC5652691 29100297

[26] Kim SS Kim KS Han IH Kim Y Bang SS Kim JH. Proliferation of Mouse Prostate Cancer Cells Inflamed by Trichomonas vaginalis. Korean J Parasitol (2021) 59:547–56. doi: 10.3347/kjp.2021.59.6.54 PMC8721307 34974661

[27] Wang J Chen GL Cao S Zhao MC Liu YQ Chen XX. Adipogenic Niches for Melanoma Cell Colonization and Growth in Bone Marrow. Lab Invest (2017) 97:737–45. doi: 10.1038/labinvest.2017.14 28218738

[28] Diedrich JD Rajagurubandara E Herroon MK Mahapatra G Huttemann M Podgorski I. Bone Marrow Adipocytes Promote the Warburg Phenotype in Metastatic Prostate Tumors *via* HIF-1alpha Activation. Oncotarget (2016) 7:64854–77. doi: 10.18632/oncotarget.11712 PMC5323121 27588494

